# Vibrationally Assisted Direct Intersystem Crossing between the Same Charge-Transfer States for Thermally Activated Delayed Fluorescence: Analysis by Marcus–Hush Theory Including Reorganization Energy

**DOI:** 10.1021/acs.jpcb.0c10605

**Published:** 2021-03-04

**Authors:** Illia E. Serdiuk, Michał Mońka, Karol Kozakiewicz, Beata Liberek, Piotr Bojarski, Soo Young Park

**Affiliations:** † Faculty of Mathematics, Physics and Informatics, University of Gdańsk, Wita Stwosza 57, 80-308 Gdańsk, Poland; ‡ Faculty of Chemistry, University of Gdańsk, Wita Stwosza 63, 80-308 Gdańsk, Poland; § Center for Supramolecular Optoelectronic Materials, Department of Materials Science and Engineering, Seoul National University, 1 Gwanak-ro, Gwanak-gu, 151-744 Seoul, Republic of Korea

## Abstract

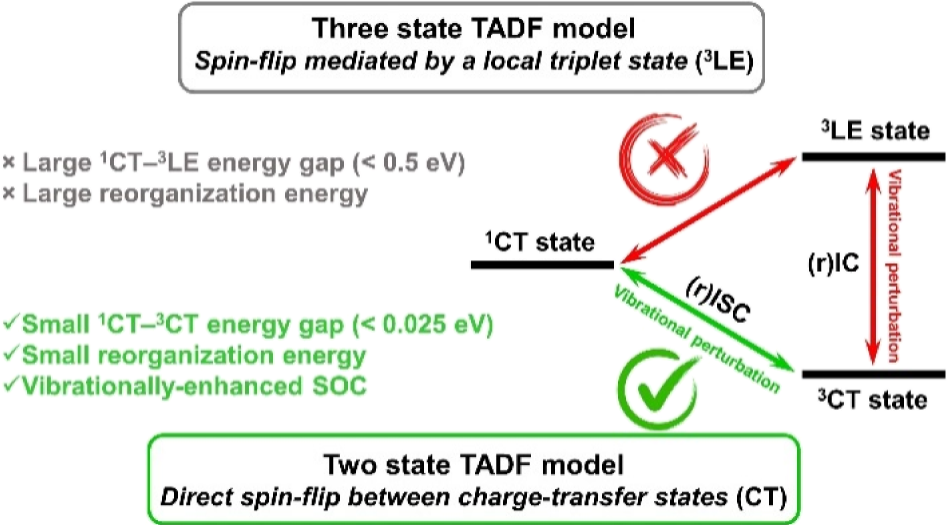

Thermally activated delayed fluorescence (TADF) has recently become an extensively investigated phenomenon due to its high potential for application in organic optoelectronics. Currently, there is still lack of a model describing correctly basic photophysical parameters of organic TADF emitters. This article presents such a photophysical model describing the rates of intersystem crossing (ISC), reverse ISC (rISC), and radiative deactivation in various media and emphasizing key importance of molecular vibrations on the example of a popular TADF dye 9,10-dihydro-9,9-dimethyl-10-(4-(4,6-diphenyl-1,3,5-triazin-2-yl)phenyl)-acridine (DMAC-TRZ). The presented experimental and theoretical investigations prove that ISC and rISC can occur efficiently between the singlet and triplet states of the same charge-transfer nature (^1^CT and ^3^CT, respectively). In emitters with the orthogonal donor and acceptor fragments, such spin-forbidden ^1^CT ↔ ^3^CT transitions are activated by molecular vibrations. Namely, the change of dihedral angle between the donor and the acceptor affords reasonable spin–orbit coupling, which together with a small energy gap and reorganization energy enable ^1^CT ↔ ^3^CT transition rates reaching 1 × 10^7^ s^–1^. Evidence of direct ^1^CT ↔ ^3^CT spin-flip and negligible role of a second triplet state, widely believed as a key parameter in the design of (r)ISC materials, change significantly the current understanding of TADF mechanism. In authors’ opinion, photophysics, and molecular design principles of TADF emitters should be revised considering the importance of vibrationally enhanced ^1^CT ↔ ^3^CT transitions.

## Introduction

Over the last decade, fast development of all-organic optoelectronics with an increasing number of potential applications provided a plethora of experimental solutions on the (sub)molecular level. In one of the most promising applications of all-organic light-emitting diodes (OLEDs), these solutions are mainly focused on the problem of triplet harvesting. Due to spin statistics, recombination of charges in organic semiconductors leads to formation of singlet (25%) and triplet (75%) excitons. In heavy-atom free molecular systems, triplet excitons usually cannot ensure fast rates of light emission and should be converted to the singlet ones for high electroluminescence efficiency.

Currently, probably the best solution for triplet harvesting is the use of materials with fast reverse intersystem crossing (rISC). This can be realized either by emitters exhibiting thermally activated delayed fluorescence (TADF) dispersed in common OLED hosts^1^ or by common fluorescent emitters dispersed in TADF emitters in the role of hosts, the so-called “hyperfluorescence” approach.^2^ Numerous photo- and electroluminescent investigations revealed strong dependence of internal and external quantum efficiencies on the rISC rate. The importance of materials with fast rISC for further development of optoelectronics cannot be thus overestimated.

Up to the moment, there has been no general theoretical model which would correctly describe the basic photophysical parameters of TADF materials such as rates of intersystem crossing (ISC), rISC, and radiative deactivation in various media. Obviously, this impedes correct understanding of the mechanism and design principles of efficient TADF materials. In this article, we present such a model and on the example of one of the most popular TADF emitters explain how high rISC and ISC rates are achieved under various conditions. To date, all organic emitters with superior rISC rates are built of spatially separated strong electronic donor and acceptor molecular fragments, which is the key principle of design strategy of TADF emitters. Such a structure enables formation of charge-transfer (CT) states, which represent the lowest excited singlet ^1^CT and triplet ^3^CT electronic states. Efficient separation of the donor and acceptor provides a negligible overlap of highest occupied molecular orbital (HOMO) and lowest unoccupied molecular orbital (LUMO), which decreases the exchange and energy gap (Δ*E*_^1^CT–^3^CT_) between the ^1^CT and ^3^CT states. All theoretical models developed before to describe the bases of TADF photophysics follow the selection rule stating that spin-flip between the ^1^CT and ^3^CT states is forbidden due to their identical nature. In fact, state-of-the-art methods of quantum chemistry predict a zero spin–orbit coupling constant (SOCME) for the ^1^CT and ^3^CT interaction. For this reason, TADF efficiency is believed to be strongly dependent on the presence of at least one additional triplet state with close energy to the ^1^CT and ^3^CT ones. This can be either a locally excited ^3^LE state localized on a donor or an acceptor^3^ or another CT state formed via electronic transition on different molecular orbitals.^4^ According to this, rISC is mediated by this third state because the transition between it and the ^1^CT state is more allowed due to their different nature. The presence of such a third state is believed as another key requirement in the design of TADF emitters.

Among the above-described cases, the one relying on the energetically close donor or acceptor ^3^LE state has been used to explain the majority of TADF examples including a triazine derivative 9,10-dihydro-9,9-dimethyl-10-(4-(4,6-diphenyl-1,3,5-triazin-2-yl)phenyl)-acridine (DMAC-TRZ) investigated here. It should be emphasized that such a three-state model predicts maximum rISC rate when ^3^LE and ^3^CT/^1^CT states are energetically close. In terms of spectroscopic parameters measured experimentally, this means that the energy gap between ^1^CT and ^3^LE states (Δ*E*_^1^CT–^3^LE_) should be zero. More advanced variations of the three-state model specify an additional condition for maximal rISC: vibronic coupling between ^3^LE and ^3^CT states.^5^ Similar criterion is found in another theoretical model, which assumes that the T_1_ state of various *s*-triazine and benzonitrile emitters is not of pure CT-nature but contains various portions of LE nature.^6^ Such a model thus suggests different nature of S_1_ and T_1_ states, which provides relatively high SOCME values and in such a way explains efficient rISC. The extent of LE contribution can be substantial in a non-polar medium, when CT energy is close to the triplet energy of a separate acceptor or donor, but it should decrease when CT is stabilized in high-polarity media. As follows from all these models, when the lowest CT states are stabilized and the Δ*E*_^1^CT–^3^LE_ energy gap increases by module, the rISC rate should decrease sharply. What is more, the same conclusion should be true for the forward ISC transition because it should also strongly depend on Δ*E*_^1^CT–^3^LE_. Here, we provide evidence that such conclusions on the dependence of ISC and rISC rates on the Δ*E*_^1^CT–^3^LE_ value are not supported by the experiment.

Some previous articles have already pointed at some discrepancies between the three-state model and the experimental behavior of TADF emitters. Namely, some of the investigations evidence the key role of molecular vibrations in TADF photophysics. For example, introduction of methyl groups at positions 1 and/or 9 of phenothiazine donor decreases drastically TADF rate and efficiency of phenothiazine-dibenzothiophene-*S*,*S*-dioxide emitters even in liquid solutions.^7^ In this case, in spite of low Δ*E*_^1^CT–^3^LE_ value, the restriction of some undetermined molecular vibrations by methyl groups was concluded to be extremely important for rISC: the more the vibrational freedom was restricted, the less TADF efficiency was observed. In the case of less flexible derivatives with more bulky isopropyl or *tert*-butyl groups, TADF was almost absent and the T_1_ state deactivated mainly via room-temperature phosphorescence. Next, it was found that the explanation of the radiative rate of TADF emitters with orthogonal donor and acceptor fragments is not possible within “frozen” optimized geometry because of zero oscillator strength predicted by calculations.^8^ It was proved that molecular vibrations should be taken into account for adequate prediction of oscillator strength and electronic transition moment. In another recent investigation, the behavior of DMAC-TRZ in solid solutions was explained by the inhomogeneity of its geometry caused by rotational isomerism and distribution of CT states.^9^ Another recent theoretical model assumed that both spin–orbit coupling (SOC, T_2_/T_1_ → S_1_) and the so-called direct SOC (T_1_ → S_1_) play important roles in rISC.^10^ However, the direct T_1_ → S_1_ transition was predicted to be efficient when the nature of T_1_ and S_1_ states is different: the presented calculations predicted 92% CT nature for the S_1_ state and 95% LE nature for the T_1_ state. The proposed model can thus also be valid only in the case of the proximity of ^1^CT and ^3^LE states. Despite the fact that the above-mentioned investigations were still explained by the three-state model, these experimental results question the primary importance of minimal Δ*E*_^1^CT–^3^LE_ value for a maximum rISC rate at *T* > 0 K where molecules exist in various excited vibronic states.

The importance of molecular vibrations for efficient TADF was also suggested for the carbene–heavy-metal complexes.^11^ Fast rISC in such emitters was explained by the thermally activated rotation along the bond between the metal atom and the donor fragment, which decreased the Δ*E*_^1^CT–^3^CT_ value and facilitated direct T_1_ → S_1_ transition. Together with the reasonable SOC values^12^ due to heavy-atom effect, this resulted in high TADF rate constant (ca. 3 × 10^6^ s^–1^). Further investigations of similar compounds revealed that the energetic closeness of ^3^LE state itself does not reduce the activation energy of rISC but results in the undesired decrease of emissive rate of the S_1_–S_0_ transition.^13^

It should also be noticed that according to the three-state model, the differences between the ^1^CT and ^3^LE states should favor the interaction between them. However, different electronic structures lead to the differences in geometry. In terms of Marcus semiclassical electron-transfer theory,^6^ this means the increase of reorganization energy of ^1^CT ↔ ^3^LE transitions (λ_^1^CT↔^3^LE_) and should not increase but rather decrease their rates. The investigations presented here prove that minimization of reorganization energy is as much important as small energy gap between singlet and triplet state (Δ*E*_ST_) value for achieving high rates of ISC and rISC. Substantial differences in the reorganization energies play the decisive role in favor of ^3^CT ↔ ^1^CT transitions but not the ^1^CT ↔ ^3^LE ones.

In this research, to check the widely accepted importance of ^3^LE state for spin-flip transitions, both Δ*E*_^1^CT–^3^CT_ and Δ*E*_^1^CT–^3^LE_ values were scanned experimentally. One of the most simple and basic but reliable spectroscopic experiments was conducted—solvatochromic measurements. The use of media of different polarities provides information on the effect of alignment of energy levels on all basic photophysical parameters, which is especially valuable for understanding TADF photophysics. DMAC-TRZ emitter was selected, which together with its various derivatives exhibits one of the best photo- and electroluminescent characteristics extensively studied lately.^14,15^ Due to the high transition dipole moment of the ^1^CT and ^3^CT states, their energies were effectively decreased with increasing medium polarity. Localized transitions are much less sensitive to the polarity of medium; therefore, the ^3^LE value is concerned as a constant. Thus, manipulation of solvent polarity, in a common notion meaning overall solvation capability, allowed us to tune Δ*E*_^1^CT–^3^LE_ from positive (low polarity, *E*_^1^CT_ > *E*_^3^LE_) to negative (higher polarity, *E*_^1^CT_ < *E*_^3^LE_) values. Importantly, evidence was found that the energy gap between ^1^CT and ^3^CT states also changes with polarity. In spite of experimental simplicity of solvatochromic experiment, it remains a challenging task for calculations using the methods of time-dependent density functional theory (TDDFT). For this reason, the results of calculations were treated here with special attention and analyzed only in the case of good correlation with experimental data.

## Methods

DMAC-TRZ was synthesized as reported previously^16^ and purified by sublimation in vacuum. Solvents for photophysical measurements were of spectroscopic grade or higher. All the photophysical measurements were conducted in the argon atmosphere.

Steady-state photoluminescence spectra were obtained with a PTI QuantaMaster 40 spectrofluorometer. All the emission spectra were corrected on the photodetector sensitivity. Photoluminescence quantum yields (PLQY) were obtained using 9,10-diphenylanthracene in cyclohexane (93%) as a reference.^17^ Time-dependent emission measurements at room temperature were obtained using a FluoTime 300 fluorescence lifetime spectrometer equipped with a LDH-P-C-375 laser head. The parameters of photophysical processes were calculated using equations described in the literature;^18,19^ for details, see the Supporting Information.

### Quantum-Chemical Calculations

The unconstrained geometry optimizations of DMAC-TRZ were performed for the ground (S_0_), excited singlet (S_1_), and triplet (T_1_, T_2_, and T_3_) electronic states at DFT/TDDFT level of theory^20^ using the Gaussian 16 program package.^21^ The B3LYP^22^ hybrid functional was used with the cc-pVDZ basis set. Nature of states (CT or LE) was established on the basis of analysis of molecular orbitals involved in each transition, for details, see the Supporting Information, Scheme S1. The energies of ^1^CT–S_0_, ^1^CT–^3^CT, and ^1^CT–^3^LE transitions were obtained by single-point calculations using the optimized geometry of ^1^CT state (S_1_); the respective values for the ^3^CT–S_0_, ^3^CT–^1^CT and ^3^LE–S_0_, ^3^LE–^1^CT transitions were obtained by single-point calculations using the optimized geometries of ^3^CT (T_1_) and ^3^LE (T_2_), respectively. Computational Δ*E*_^1^CT–^3^CT_ and Δ*E*_^1^CT–^3^LE_ values represent differences between minimal energies of respective states. The electronic energies and transition energies of rotational isomers in ^1^CT, ^3^CT, and ^3^LE states were calculated by single-point calculations via dihedral angle scan using the geometries of the respective states. The SOC constants were calculated within the ORCA program package,^23^ using ZORA relativistic contraction of the TZVP basis set.

For each rotational isomer, the rate constants of ISC and rISC via ^1^CT → ^3^CT, ^1^CT → ^3^LE and ^3^CT → ^1^CT, ^3^LE → ^1^CT transitions were calculated using the Marcus–Hush equation
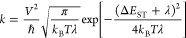
1where *k* is a rate constant, *V* is the SOC constant, λ is the sum of internal and external (λ_solv_) reorganization energies for the respective transition, Δ*E*_ST_ is an energy gap between ^1^CT and the respective triplet state, *k*_B_ is the Boltzmann constant, h̵ is the reduced Planck’s constant, and *T* is the temperature (298.15 K). For detailed procedures and examples of calculations, see the Supporting Information.

## Results and Discussion

**Figure 1:**
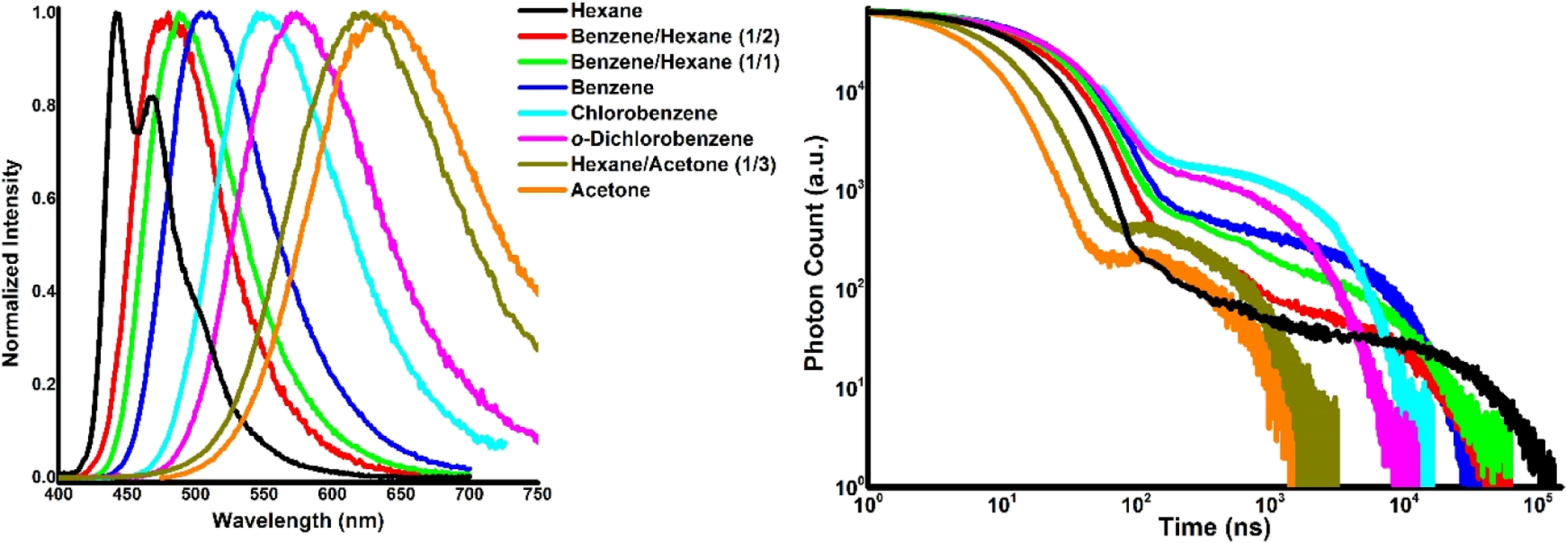
Intensity-normalized emission spectra and decays of DMAC-TRZ in various solutions.

Figure 1 shows the emission spectra and decays, respectively, of DMAC-TRZ in various liquid solutions. Strong positive solvatochromism evidenced energy decrease of the ^1^CT state with the increase of medium polarity. Taking into account the 440 nm onset of ^3^LE phosphorescence measured in frozen methylcyclohexane solution,^16^ the Δ*E*_^1^CT–^3^LE_ values in these experiments varied from +0.1 eV in hexane to −0.4 eV in acetone. In further discussion, to analyze the medium effect and avoid fluctuations of composition of solvent mixtures during the removal of oxygen, onset of fluorescence spectrum (*E*_^1^CT_) is used as an internal polarity parameter.

**Figure 2:**
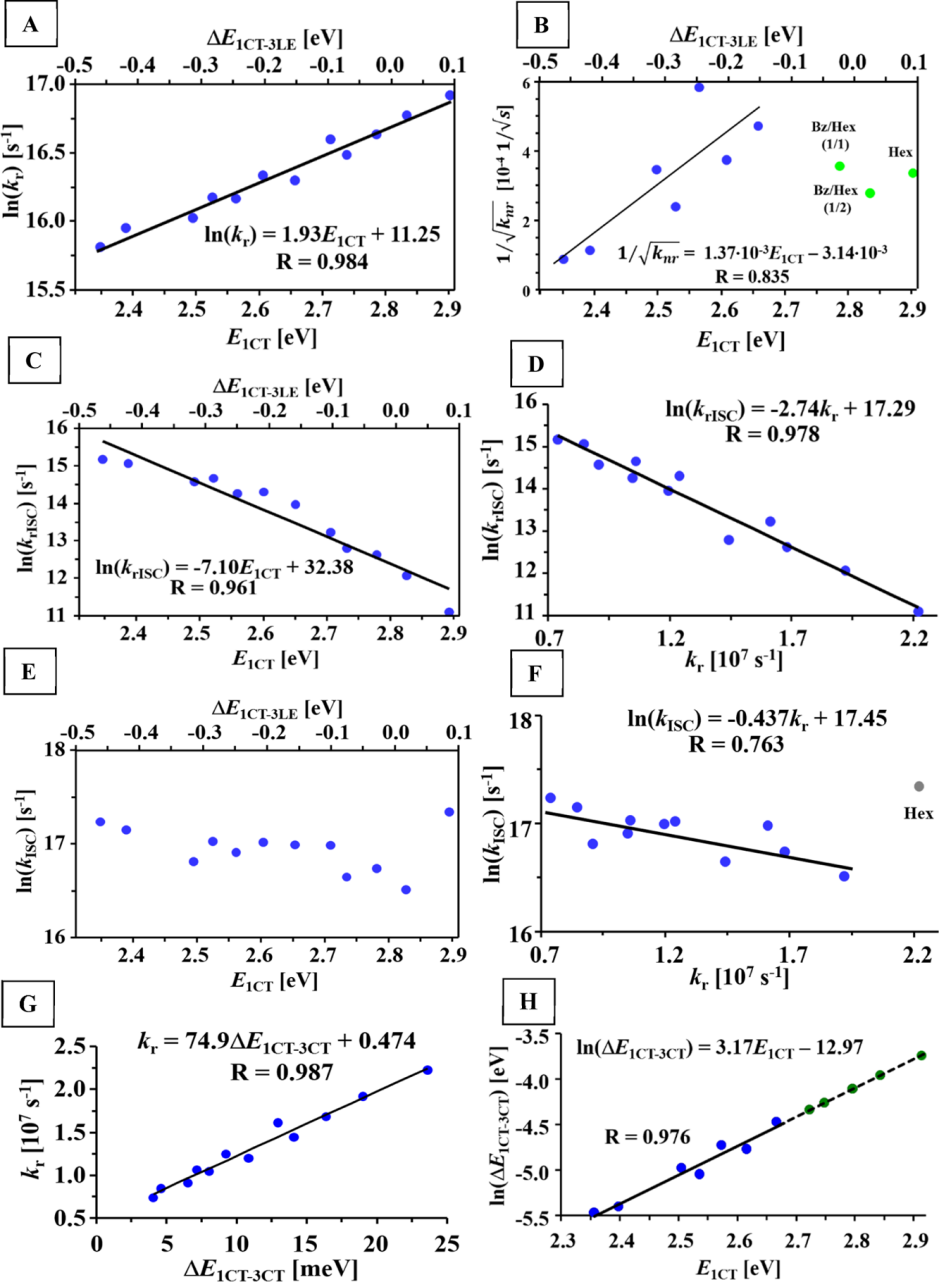
Dependencies of experimental rate constants of radiative (A) and nonradiative (B) deactivation, rISC (C) and ISC (E) on the energy of ^1^CT state and Δ*E*_^1^CT–^3^LE_ energy gap. Dependencies of experimental rate constants of rISC (D) and ISC (F) on radiative rate constant. Correlation between the experimental rate constant of radiative deactivation and reconstructed ^1^CT–^3^CT energy gap (G). Logarithm of energy gap between ^1^CT and ^3^CT states as a function of ^1^CT-state energy (H, blue points obtained using experimental *k*_rISC_ values, green ones calculated by extrapolation).

Rates and yields of photophysical processes are presented in Table S1 (Supporting Information). The rate constant of radiative deactivation (*k*_r_) exhibited strong exponential dependence on *E*_^1^CT_ (Figure 2A). The highest *k*_r_ value of 2.2 × 10^7^ s^–1^ was obtained for an *E*_^1^CT_ of 2.91 eV in non-polar hexane, while the lowest *k*_r_ of 7.4 × 10^6^ s^–1^ was obtained for an *E*_^1^CT_ of 2.36 eV in polar acetone. Such a reduction of *k*_r_ with the increase of polarity is well known for donor–acceptor molecular systems.^24^ In current discussion, it should be noted that the observed dependence provides evidence that the increasing polarity and solvent-relaxation efficiency in the excited state favors more effective separation of frontier orbitals involved in the S_1_–S_0_ transition. On the one hand, this reduces oscillator strength and *k*_r_. On the other hand, this favors minimization of the exchange energy between S_1_ and T_1_ states of the same nature, which leads to the reduction of Δ*E*_^1^CT–^3^CT_ value. Within the same D–A chromophore, *k*_r_ can thus be referred as a measure of Δ*E*_^1^CT–^3^CT_. This assumption is quite important because of the lack of reliable experimental methods for the estimation of Δ*E*_^1^CT–^3^CT_ and ^3^CT-state energy, especially in solutions and at room temperature. As will be discussed further, the determined Δ*E*_^1^CT–^3^CT_ in fact correlates perfectly with *k*_r_.

Analysis of the emission decay profiles (Figure 1) leads to important conclusions on the polarity effect on nonradiative transitions. When polarity increases from hexane to *o*-dichlorobenzene, the time range in which emission occurs is shortened, but the intensity of the delayed component represented by the intensity of the second decay plateau is increased. This indicates the increase of rISC rate with medium polarity. In more polar acetone and 75% acetone in hexane, the time range of emission shortens sharply, indicating strong increase of nonradiative deactivation. In terms of the determined parameters (Table S1, Supporting Information), in a low-polarity medium, the rate constant of nonradiative deactivation (*k*_nr_) of ^1^CT state is minimal, when Δ*E*_^1^CT–^3^LE_ is close to zero. One can thus suggest that the energetic closeness of ^1^CT and ^3^LE states is important for high PLQY. In more polar solvents, lower ^1^CT level corresponds to faster nonradiative deactivation. Importantly, the increase of *k*_nr_ is the key factor decreasing PLQY in polar media but not the changes in ISC or rISC rates. In polar media, the main nonradiative deactivation channel is most likely internal conversion, the rate constant of which is proportional to 1/Δ*E*_S_1_–S_0__^2^.^25^ In fact, *k*_nr_ shows good correlation with 1/Δ*E*_S_1_–S_0__^2^ in polar media (Figure 2B), but not in the low-polar one, indicating different mechanisms of nonradiative deactivation probably due to slow rISC.

### rISC Rate

**Figure 3:**
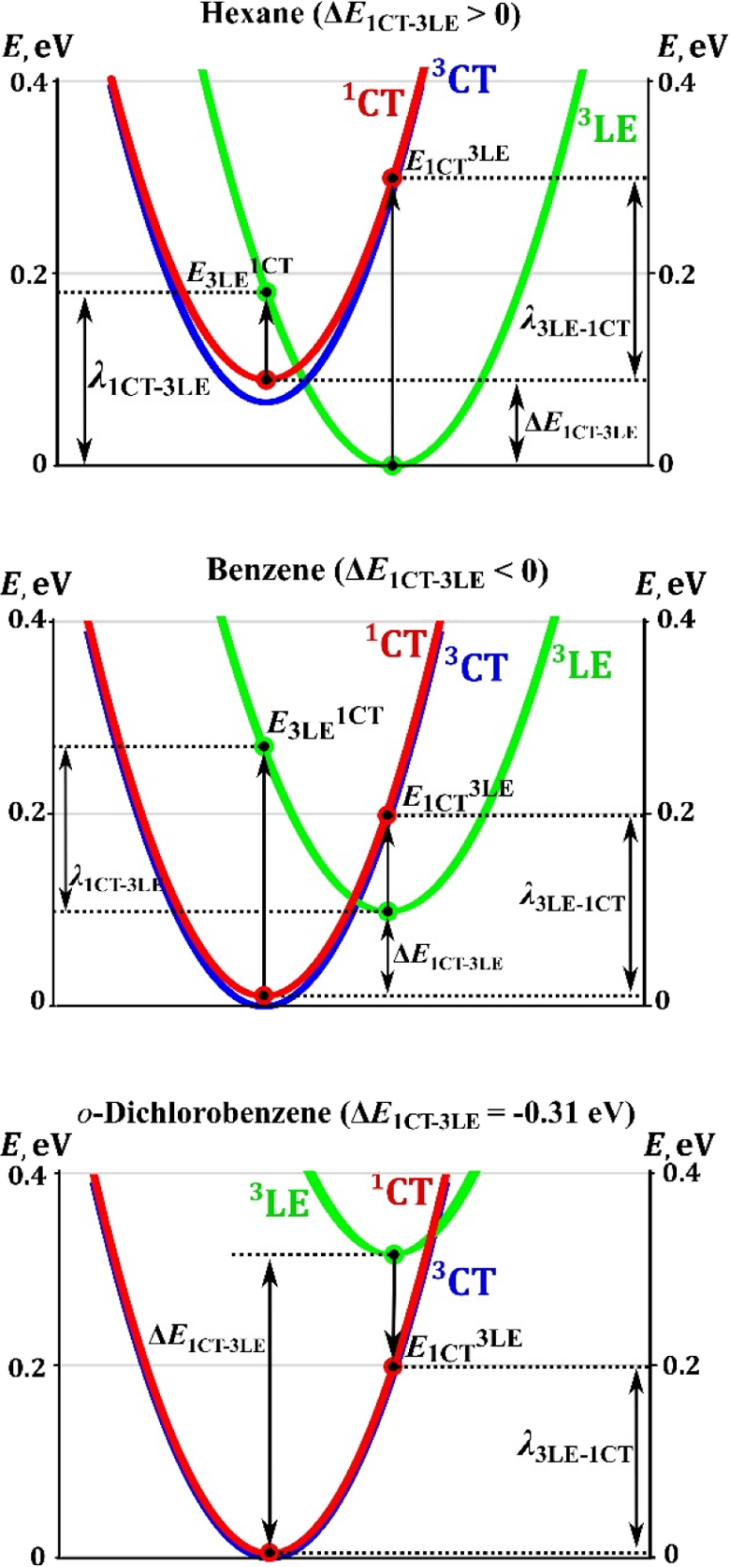
Potential energy curves of the lowest excited states in various media.

To present further evidence of Δ*E*_^1^CT–^3^CT_ dependence on polarity, rISC is discussed prior to ISC. The value of *k*_rISC_ shows strong logarithmic dependence on polarity of medium, namely, *E*_^1^CT_ as a polarity parameter: the lower the *E*_^1^CT_, the higher the ln(*k*_rISC_) (Figure 2C). In terms of the Δ*E*_^1^CT–^3^LE_ energy gap, rISC reaches highest rate when ^1^CT is most separated from the ^3^LE state. In contrast to the three-state model, experimental data thus do not support maximal rISC rate at the point of energetic degeneracy of CT and locally excited states. When *E*_^1^CT_ decreases, the alignment of excited states changes (Figure 3): ^3^CT becomes the lowest triplet state and the fraction of excited molecules in the second triplet state (^3^LE) decreases sharply. Despite that Δ*E*_^1^CT–^3^LE_ becomes negative increasing the driving force of ^3^LE → ^1^CT transition, decreased population of the ^3^LE state should result in *k*_rISC_ decrease, which is not the case.

Figure 3 illustrates the significance of reorganization energy λ_^1^CT↔^3^LE_ and driving force Δ*E*_^1^CT–^3^LE_ for the transitions between the ^1^CT and ^3^LE states of different geometries. Within the Marcus theory, the maximum rate of ^3^LE → ^1^CT transition is not predicted for the same energy of the ^1^CT and ^3^LE states because of the additional reorganization energy involved. If rISC occurs via the ^3^LE → ^1^CT transition, the dependence of *k*_rISC_ on *E*_^1^CT_ should contain two regions. First is “normal region”, where −Δ*E*_^1^CT–^3^LE_ < λ_^1^CT↔^3^LE_ (Figure 3, hexane and benzene). In this region, the rISC rate should increase, when −Δ*E*_^1^CT–^3^LE_ draws closer to λ_^1^CT↔^3^LE_ and reach maximum, when these values are equal. In the second region, “inverted region”, where −Δ*E*_^1^CT–^3^LE_ > λ_^1^CT↔^3^LE_, the rISC rate should decrease as the −Δ*E*_^1^CT–^3^LE_ value increases (Figure 3, *o*-dichlorobenzene). Clearly, these regions are not observed in the experimental *k*_rISC_ on *E*_^1^CT_ dependence.

On the other hand, the experimental rISC rate shows strong exponential dependence on *k*_r_ (Figure 2D), which as suggested above correlates with the energy gap between the CT states of different multiplicity. At this point, it is concluded that *k*_rISC_ is strongly dependent on Δ*E*_^1^CT–^3^CT_ but not the Δ*E*_^1^CT–^3^LE_ value.

**Figure 4:**
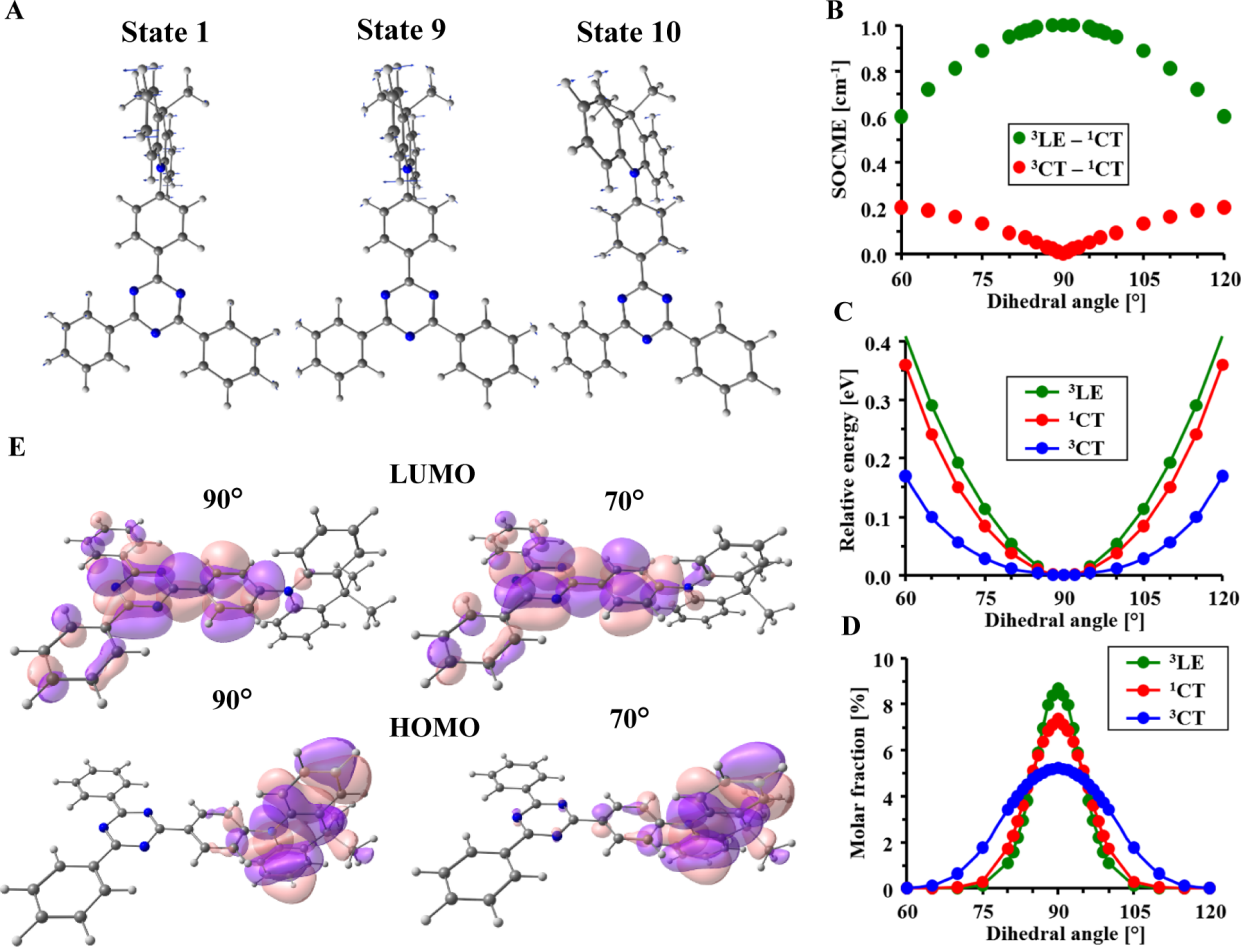
(A) Vectors of atomic movements in selected lowest vibrational states of the ^1^CT state. (B) Dependence of SOCME between ^3^LE–^1^CT (^3^LE geometry) and ^3^CT–^1^CT (same for the ^3^CT and ^1^CT geometries) states on the dihedral angle between donor and acceptor fragments. (C) Dependence of the relative energy of excited state on the dihedral angle. (D) Dependence of the molar fractions of rotational isomers at room temperature. (E) HOMO and LUMO calculated for various torsion angles between DMAC and TRZ fragments (contour value 0.017, ^1^CT-state geometry).

To explain the experimental dependences, the effect of specific molecular vibrations on the electronic features of DMAC-TRZ is analyzed. As was mentioned above, in the optimal geometries with ideally orthogonal DMAC and TRZ fragments, SOCME between ^3^CT and ^1^CT states is 0.00 cm^–1^ which excludes the possibility of efficient spin-flip transition. It is however well known that at temperatures above 0 K, molecules exist in the excited vibronic states. Molecular vibrations are responsible for the violation of some selection rules such as symmetry forbidden and n−π* transitions.^26^ Similarly, the transitions forbidden by the El-Sayed rule, based on the electronic nature of states, can occur due to vibrational coupling perturbations. Apparently, a theoretical model describing photophysics of TADF emitters should take into account the effect of molecular vibrations on geometry. In the first vibronic mode of DMAC-TRZ, the dihedral angle (θ) between the donor and acceptor is changing (Figure 4A). Such molecular vibrations have immense influence on electronic properties, and therefore, the model presented below is based on these vibrations.

Importantly, SOC between the ^3^CT and ^1^CT states was found to be extremely sensitive to the dihedral angle θ (Figure 4B): SOCME is zero in optimal geometry with θ of 90°, rises up to 0.05 cm^–1^ at 85°, and reaches 0.2 cm^–1^ when θ is below 65°. The discussed vibrational mode has lower energy in the ^3^CT state (9.84 cm^–1^) as compared to ^1^CT (13.20 cm^–1^). Consequently, the energies (Figure 4C) and molar fractions (Figure 4D) of rotational isomers are also different in the ^1^CT and ^3^CT states. In the ^1^CT state, rotamer energy increases by 0.2 eV when θ reaches 67 or 113°, while for the ^3^CT state, this value is 58° (122°). For this reason, mean SOCME values of 0.043 and 0.051 cm^–1^ are obtained for the ^1^CT → ^3^CT and ^3^CT → ^1^CT transitions, respectively. The increase of SOC under deviation from orthogonality is explained by the increasing overlap of molecular orbitals involved in CT transitions, namely, increased contribution of the phenyl-*s*-triazine fragment in HOMO (Figure 4E).

The same analysis conducted for the ^3^LE → ^1^CT transition in the ^3^LE-state geometry revealed that the SOCME value between these two states is 1.0 cm^–1^. This value is very close to most of other organic TADF systems explained so far via the three-state model.^5,6,10,14,15^ The SOC of the ^3^LE → ^1^CT transition in DMAC-TRZ decreases when the torsional angle θ deviates from 90° (Figure 4B). Higher energy of the discussed rotation also results in lower contribution of the rotational isomers than in the case of CT state (Figure 4D).

Prior to the theoretical prediction of rISC rate using the Marcus–Hush equation (see Methods), the effect of dihedral angle θ on Δ*E*_^1^CT–^3^CT_ and reorganization energy should be analyzed. Expectedly, deviation from orthogonal geometry and increase of HOMO–LUMO overlap cause the increase of Δ*E*_^1^CT–^3^CT_. When θ = 90°, TD-DFT/B3LYP calculations predict the Δ*E*_^1^CT–^3^CT_ value of 6.6 meV, which rises up gradually to 0.20 eV at 60° (Figure S1, Supporting Information). Within the Marcus theory, internal (structural) and external (solvent) reorganization energies are distinguished. For the ^3^CT → ^1^CT transition, calculations predict very low structural reorganization energy <0.5 meV, which is independent of θ within 90–65°. Due to a very similar geometry and electronic parameters of these two states, solvent reorganization energy (λ_solv_) should also be low. One can expect that similar to Δ*E*_^1^CT–^3^CT_, the λ_solv_ value should increase together with the growing difference between the electronic parameters of the ^3^CT and ^1^CT states. In further calculations, we thus assumed that the sum of reorganization energies for ^3^CT ↔ ^1^CT transitions (λ_^1^CT–^3^CT_) is equal to Δ*E*_^1^CT–^3^CT_.

The rISC rate constant calculated as a statistical sum of the rate constants of ^3^CT → ^1^CT transition for various rotational isomers using the Marcus–Hush equation and calculated Δ*E*_^1^CT–^3^CT_ and SOCME values (Table S2, Supporting Information) gives a *k*_^3^CT→^1^CT_ value of 2.26 × 10^6^ s^–1^. This value matches perfectly the experimental *k*_rISC_ value of 2.1 × 10^6^ s^–1^ in *o*-dichlorobenzene. The predicted S_1_–S_0_ transition maximum of 611 nm is also in good correlation with the experimental emission maximum in *o*-dichlorobenzene at 570 nm.

Such a remarkable match of experimental and computational data leads to two main conclusions. First, the B3LYP calculations of D–A-type emitters correlate very well with the experiment in the medium of relatively high polarity. The most important conclusion is that the ^3^CT → ^1^CT transition can solely afford high rISC rates due to molecular vibrations. High rate of ^3^CT → ^1^CT transition is a combination of couple of factors. First is vibrationally activated SOC between the same CT states of different multiplicities. Even though it is 10–20 times smaller than the SOC between the ^3^LE and ^1^CT states, the small Δ*E*_^1^CT–^3^CT_ energy gap together with almost identical geometry and thus comparably small reorganization energy enable fast ^3^CT → ^1^CT rate. As discussed further, the reorganization energy of ^3^LE → ^1^CT transition is much larger, which is a decisive factor in favor of ^3^CT → ^1^CT transition.

Similar analysis of the experimental *k*_rISC_ in polar media (*E*_^1^CT_ ≤ 2.67 eV) was done. Under such conditions, according to the Boltzmann distribution, the population of ^3^LE state is below 0.2% and thus ^3^LE → ^1^CT transition cannot contribute considerably to rISC. The ^3^CT → ^1^CT transition is thus the only pathway of rISC and its parameters can be estimated. The Δ*E*_^1^CT–^3^CT_ values corresponding to experimental *k*_rISC_ according to the Marcus–Hush equation are presented in Table S1 (Supporting Information). The thus-obtained Δ*E*_^1^CT–^3^CT_ values decrease with the rise of medium polarity, which supports perfectly the conclusions drawn above based on the experimental *k*_r_ values (Figure 2G). The dependence of Δ*E*_^1^CT–^3^CT_ on *E*_^1^CT_ is best fitted by a logarithmic function (Figure 2H). Its extrapolation afforded the Δ*E*_^1^CT–^3^CT_ values in less polar media. Consequently, the lowest energy gap of 4.2 meV is obtained for the most polar acetone solution, while the highest value of 24 meV corresponds to the hexane solution.

**Figure 5:**
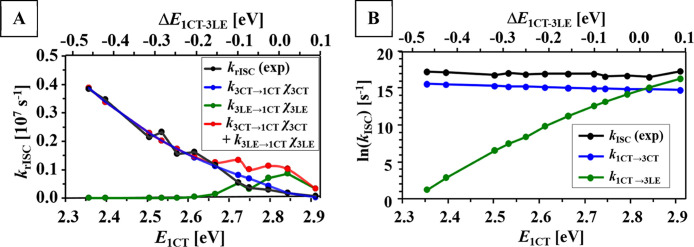
Comparison of calculated (Table S3) and experimental (Table S1) rates of triplet–singlet transitions (A) and singlet–triplet transitions in logarithmic scale (B) in a function of ^1^CT-state energy. The rate constants of ^3^LE ↔ ^1^CT transitions were calculated using λ_solv_ = 0.3 eV.

The *k*_^3^CT→^1^CT_ values calculated using the reconstructed dependence of Δ*E*_^1^CT–^3^CT_ on *E*_^1^CT_ are compared with the experimental *k*_rISC_ in Figure 5A. To take into account the medium-dependent population of species in ^3^CT and ^3^LE states, *k*_^3^CT→^1^CT_ is multiplied by the molar fraction of molecules in the ^3^CT state at 298 K (χ_^3^CT_).

In the media of lower polarity with *E*_^1^CT_ ≥ 2.72 eV, the molar fractions of species in the ^3^LE state should be taken into account (Table S3, Supporting Information). To calculate the rate of ^3^LE → ^1^CT transition, the experimental Δ*E*_^1^CT–^3^LE_ and computationally predicted structural reorganization energies (λ_^3^LE–^1^CT_, 0.209–0.226 eV) for various rotational isomers were used (for detailed procedure, see the Supporting Information). The thus-obtained *k*_^3^LE→^1^CT_ (λ_solv_ = 0.3 eV) multiplied by the molar fraction of molecules in the ^3^LE state at 298 K (χ_^3^LE_) are plotted in Figure 5A (for calculations using lower λ_solv_, see the Supporting Information). According to this, the ^3^LE → ^1^CT transition should have the key contribution to rISC in the region of low polarity with *E*_^1^CT_ close to 2.8 eV and Δ*E*_^1^CT–^3^LE_ close to zero. In this region, the three-state model predicts maximum rISC rate, which is well described by the dependence of calculated *k*_^3^LE→^1^CT_ on *E*_^1^CT_. This however completely contradicts our experimental data: *k*_rISC_ do not reach any maximum at Δ*E*_^1^CT–^3^LE_ ≈ 0. In contrast, the experimental dependence of *k*_rISC_ on *E*_^1^CT_ is almost perfectly described by the rates of ^3^CT → ^1^CT transition, but by neither the ^3^LE → ^1^CT one nor the sum of rates of these two transitions even in the low-polarity region (Figure 5A). Therefore, the data presented above unambiguously prove the decisive role of ^3^CT → ^1^CT transition, but not the ^3^LE → ^1^CT one in the rISC process in DMAC-TRZ.

### ISC Rate

ISC is another important process representing forward transformation of singlet S_1_ state to the triplet ones. ISC and rISC are closely connected; thus, any theoretical model which explains rISC but fails to describe correctly ISC cannot be regarded as satisfactory.

The experimental ISC rate shows complex dependence on the ^1^CT-state energy (Figure 2E). In hexane (*E*_^1^CT_ = 2.91 eV), an ISC rate constant (*k*_ISC_) value of 3.4 × 10^7^ s^–1^ is the highest, but it decreases sharply to 1.5 × 10^7^ s^–1^ in the benzene–hexane (1:2, v/v) mixture, where the *E*_^1^CT_ value is 2.84 eV. A further decrease of *E*_^1^CT_ causes gradual increase of *k*_ISC_ up to 3.1 × 10^7^ s^–1^ in acetone. According to the alignment of excited states, the decrease of *E*_^1^CT_ in polar media makes the ^1^CT → ^3^LE transition endothermic (Figure 3). Under such a distancing of ^1^CT and ^3^LE states, if the ^1^CT → ^3^LE transition was the main pathway of ISC, a sharp decrease of *k*_ISC_ with the Δ*E*_^1^CT–^3^LE_ value would be observed, which is not the case.

In contrast, *k*_ISC_ grows exponentially with the decrease of *k*_r_ (Figure 2F), regarded as a measure of reduction of the ^1^CT–^3^CT energy gap. These findings provide evidence that when the ^1^CT and ^3^CT states have lower energy than the ^3^LE one, ISC occurs mainly via the ^1^CT → ^3^CT channel in the nanosecond regime, even though it is forbidden by the El Sayed’s rules.

Similar to rISC, two mechanisms of ISC in DMAC-TRZ can be suggested: ^1^CT → ^3^LE and ^1^CT → ^3^CT transitions. In hexane, the ^1^CT → ^3^LE transition rate calculated using the Marcus–Hush equation is close to experimental *k*_ISC_. However, in more polar media, *k*_^1^CT→^3^LE_ decreases sharply (Figure 5B) due to the increasing negative value of Δ*E*_^1^CT–^3^LE_ and thus growing endothermicity of this process. For example, in acetone and *o*-dichlorobenzene, *k*_^1^CT→^3^LE_ does not exceed 3 and 1 × 10^3^ s^–1^, respectively, and thus, its contribution to ISC is negligible. On the other hand, the rate constant of ^1^CT → ^3^CT transition calculated as a statistical sum of *k*_^1^CT→^3^CT_ of various rotational isomers correlates well with the experimental *k*_ISC_ in all the investigated media except for hexane: *k*_^1^CT→^3^CT_ increases with the decrease of *E*_^1^CT_ (Figure 5B). The reason of such a dependence is the above-mentioned decrease of Δ*E*_^1^CT–^3^CT_ with growing medium polarity. Therefore, in almost all experimental conditions, ISC proceeds via the ^1^CT → ^3^CT transition. Only in non-polar hexane, where the CT states are the least stabilized and Δ*E*_^1^CT–^3^CT_ reaches maximum, both pathways are realized and the ^1^CT → ^3^LE transition contributes noticeably to ISC.

In spite of perfect prediction of *k*_ISC_–*E*_^1^CT_ dependence, the calculated *k*_^1^CT→^3^CT_ values are ca. 4–5 times lower than the experimental ones. Apparently, real SOCME values of the ^1^CT → ^3^CT transition are higher. Similarly, the radiative deactivation rate calculated as the statistical sum of various rotational isomers (described in detail in the Supporting Information) is 4.2 times underestimated as compared to the experimental value in *o*-dichlorobenzene (Table S4, Supporting Information). It should be noticed that the discussed approach is a simplified model based on the first-mode vibrations. Even though these vibrations have the key influence on the electronic properties of the CT states due to the change of dihedral angle θ between donor and acceptor planes, some other vibrations of higher energies also reduce the separation of HOMO and LUMO (Figure 4A). For example, vibrations of the ninth vibronic state also change θ, and in the tenth state, the planarity of the donor is disrupted. These vibrations lead to further increase of SOC as well as oscillator strength and *k*_r_. More correct description of each TADF emitter should take into account the influence of all vibrations characteristic for each compound on the CT state. It should be noticed that, in general, understanding of vibrational enhancement of SOC cannot be simplified to the conformational changes. We, however, believe that the simplified approach presented here can be successfully used for design and semiqualitative unified description of rates of ISC, rISC, and radiative deactivation of a large number of D–A-type emitters with an orthogonal structure.

### Connection with Other Investigations

The analysis of experimental reports on other emitters proves that within similar molecular systems, the highest rISC and/or external quantum efficiency (EQE) is achieved when the CT states are most stabilized and thus, as concluded above, Δ*E*_^1^CT–^3^CT_ is minimized. This can be achieved in two ways. First is the change of medium or the host matrix. The reported experiments generally provide evidence that higher polarity favors faster rISC and, in the absence of nonradiative deactivation, higher photo- and electroluminescence efficiency. As an example, 2,7-bis(9,9-dimethyl-acridin-10-yl)-9,9-dimethylthioxanthene-*S*,*S*-dioxide (DDMA-TXO2) exhibited faster rISC in the DPEPO host than in Zeonex films,^27^ OLED devices containing tBuCzDCNPy emitter showed more than 3 times higher EQE when the DPEPO host was used instead of less polar mCP,^28^ as well as TTAZ and TXAZ emitters dispersed in the DPEPO host instead of the less polar medium, the mixture of mCP and TSPO1 (1:1) hosts.^29^

Almost all previous theoretical models were developed on the basis of experimental photophysical data in the media of low polarity such as OLED hosts (mCP, CBP, DPEPO, etc.) or polymer matrixes such as Zeonex, polystyrene, and PMMA. Within such media, the variation of *E*_^1^CT_ of a selected emitter usually does not exceed 0.15 eV, which is too small to study a case with large Δ*E*_^1^CT–^3^LE_. Another important remark: in TADF emitters bearing conjugated fragments such as phenothiazine, phenoxazine, phenyl-*s*-triazine, phenylpyrimidine, phenazine, benzophenone, naphthalimide, and so forth, as well as some carbazole derivatives and similar aromatic heterocycles, the ^3^LE energies are relatively low and, in non-polar medium, represent the T_1_ states. Numerous TADF emitters can serve as examples: PTZ-DBTO2,^30^ DPTZ-DPTO2,^31^ and their derivatives based on which the three-state model was mainly developed, numerous *s*-triazine derivatives,^14−16,29,32^ DBT-BZ-DMAC,^33^ NAI,^34^ various indolo[3,2-*b*]indole derivatives,^35^ POZ-DBPHZ,^36^ and so on. When polarity increases, the CT states of such emitters are getting energetically closer to ^3^LE, but simultaneously, the energy difference between ^1^CT and ^3^CT states also decreases, which most likely plays the key role in TADF.

The second way to change the CT-state energies is chemical modification. According to the literature reports, within derivatives bearing similar donor and acceptor fragments, better TADF parameters are observed in the emitters with higher donor/acceptor strength. For example, in toluene, DMAC-TRZ^16^ bearing strong *s*-triazine acceptor shows higher rISC rate (7 × 10^5^ s^–1^) as compared to its weaker 2-pyrimidine (no TADF) and 4-pyrimidine analogues (6 × 10^4^ s^–1^).^37^ In the DPEPO host, despite a larger absolute Δ*E*_^1^CT–^3^LE_ value, CT states of DMAC-TRZ are more stabilized and afford EQE_max_ of 25%, in contrast to the pyrimidine analogues not exceeding 8% and 12%, respectively. Similarly, the change of spiroacridine donor to a weaker phenazasiline one in combination with the same aryl-1,3,5-triazine acceptors led to destabilization of CT states, slower TADF, and lower EQE.^29^

The only one known example of a molecular emitter with an extremely small Δ*E*_^1^CT–^3^CT_ value of < 2 meV and a large Δ*E*_^1^CT–^3^LE_ value of 0.2 eV serves as an excellent proof for the key importance of energetic closeness of ^3^CT and ^1^CT (compound **2**, ref (38)). Taking into account the very low Δ*E*_^1^CT–^3^CT_ value emphasized by authors, the experimental evidence of very fast ISC and rISC exceeding the rate of radiative deactivation supports the vibrationally enhanced ^3^CT ↔ ^1^CT SOC model presented here.

As concluded above, one of the factors of efficient ^3^CT ↔ ^1^CT transformation is the vibrationally enhanced SOC with an average value of 0.05 cm^–1^. In one of the pioneering works, the phenomenon of hyperfine coupling between the ^1^CT and ^3^CT states was suggested as a main reason explaining fast population of T_1_ state in TADF emitters in time-resoled EPR experiment.^39^ According to this model, SOCME due to hyperfine interaction can however reach a maximum of 0.2 cm^–1^, when Δ*E*_^1^CT–^3^CT_ is below 0.051–0.025 meV (0.4–0.2 cm^–1^). This can be valid for radical pairs; however, in almost all known molecular TADF emitters, the Δ*E*_^1^CT–^3^CT_ value is hundred times higher. A conclusion on negligible impact of hyperfine coupling on the TADF photophysics was also made previously on the basis of theoretical modeling.^5^

Finally, the vibronically assisted ISC model was applied for the 2CzPN and 4CzIPN emitters to explain the electron spin resonance (ESP) experiments.^40^ It should be mentioned that donor and acceptor in these emitters are not completely orthogonal, which provides HOMO/LUMO overlap, differences in the S_1_ and T_1_ states’ nature, and thus non-zero SOC with large Δ*E*_ST_ already in the optimal geometry.^41^ However, the ESP results consistent with the vibronically assisted ISC model indicate that molecular vibrations play the key role in facilitating direct ISC and rISC via the S_1_ ↔ T_1_ in such compounds. Therefore, the model presented here should be valid not only for D–A emitters with orthogonal structure and well-separated frontier molecular orbitals but also for other kinds of CT TADF emitters with appropriate Δ*E*_ST_ values.

In DMAC-TRZ, substantial differences in geometries and dipole moments of ^1^CT and ^3^LE states lead to large internal and external reorganization energies for the ^1^CT ↔ ^3^LE transitions. To summarize the key numerical data for DMAC-TRZ, the calculated internal reorganization energies λ_^1^CT–^3^LE_ and λ_^3^LE–^1^CT_ are 179.6 meV and 208.8, respectively, which are more than 350 times higher than the internal λ_^1^CT–^3^CT_ value of 0.5 meV. The analysis of solvatochromic effect on the photophysical parameters revealed that the ^1^CT ↔ ^3^LE transition with large reorganization energy do not explain the experimental growth of rISC and ISC rates with the polarity of medium. In various media from hexane to acetone solutions, the Δ*E*_^1^CT–^3^LE_ values estimated experimentally changed in the large range from +94 to −460 meV, whereas the Δ*E*_^1^CT–^3^CT_ values changed from 24 to 4.2 meV. The corresponding TD-DFT calculated values were 559 and 6.6 meV, respectively. In contrast to the ^1^CT ↔ ^3^LE transitions, the ^1^CT ↔ ^3^CT ones perfectly explained the experimental dependencies due to much smaller reorganization energy and energy gap as well as vibrationally enhanced SOC.

In D–A emitters, the role of ^3^LE state in spin-flip processes should thus be carefully analyzed using both Δ*E*_^1^CT–^3^LE_ and λ_^1^CT–^3^LE_ values, namely, how the change of latter values correlate with the experimental rate constants. In our opinion, the evidence of fast ISC and rISC between the ^1^CT and ^3^CT states presented here significantly change the current understanding of TADF photophysics. Previous interpretation of the photophysical properties of TADF emitters based on the Δ*E*_^1^CT–^3^LE_ value as the key parameter and negligible rate of the ^3^CT ↔ ^1^CT transitions should be reconsidered.

## Conclusions

The main conclusion of this research is that correct description of the basic photophysical parameters of TADF emitters such as rates of ISC, rISC, and radiative deactivation is not possible without taking into account molecular vibrations and deviation from the orthogonality of D–A planes. A relatively simple and illustrative model presented here brings further important consequences. The evidence of efficient interaction of the same CT states with different multiplicities changes significantly the current understanding of TADF. Photophysics of some emitters as well as molecular design principles should be revised with an accent on Δ*E*_^1^CT–^3^CT_.

Experimental investigations of DMAC-TRZ in solvents of large range of polarity provided evidence of the increase of rates of forward and rISC under the stabilization of CT states. Supported by the TDDFT calculations, the polarity dependence of ISC and rISC was explained by the growing efficiency of the ^1^CT ↔ ^3^CT interaction, which is forbidden in “frozen” optimal excited-state geometry but is effectively activated by molecular vibrations and accelerated in polar media due to the reduction of the ^1^CT–^3^CT energy gap.

To describe correctly the experimental data, a theoretical model was developed, which treats photophysical parameters as statistical sums of respective values for selected rotational isomers existing at room temperature. Molecular vibrations changing the dihedral angle between donor and acceptor fragments were found to increase the SOC sharply between the ^1^CT and ^3^CT states of the same nature. According to the theoretical and experimental results, ISC and rISC occur through the ^3^CT ↔ ^1^CT channel exclusively. The exception is the medium of lowest polarity, hexane, where the Δ*E*_^1^CT–^3^CT_ value is the highest one and the ISC rate is abnormally high, indicating some influence of the ^3^LE state.

The main factors enabling effective ^1^CT ↔ ^3^CT transitions are the vibrationally enhanced SOC, very small energy gap, and comparably small reorganization energy due to similar geometries in the ^1^CT and ^3^CT states. In contrast, much larger reorganization energy λ_^1^CT–^3^LE_, growing |Δ*E*_^1^CT–^3^LE_| value, and decreasing population of the ^3^LE state result in negligible contribution of ^3^LE ↔ ^1^CT transition rate in media more polar than hexane.

Further development of high-precision theoretical TADF model at a given temperature should take into account numerous molecular vibrations and their impact on the electronic properties of lowest excited states. Moreover, special attention should be given to the effects of viscosity of medium on molecular vibrations, especially in the case of amorphous emitting layers in optoelectronic devices.

## Supporting Information Available

Experimental photophysical parameters and detailed procedure of their determination, results of DFT calculations, detailed procedures, and examples of calculations of ISC, rISC, and *k*_r_ within the developed model (PDF)
