# Supplementary material for: Vibrationally Assisted Direct Intersystem Crossing between the Same Charge-Transfer States for Thermally Activated Delayed Fluorescence: Analysis by Marcus–Hush Theory Including Reorganization Energy
Source: J Phys Chem B. 2021 Mar 4;125(10):2696–706. doi: 10.1021/acs.jpcb.0c10605 (PMC8028332; doi:10.1021/acs.jpcb.0c10605)
Supplement: Supplementary file 1 — jp0c10605_si_001.pdf [file jp0c10605_si_001.pdf]

SUPPLEMENTAL INFORMATION

Vibrationally Assisted Direct Intersystem Crossing Between the Same Charge Transfer States for Thermally Activated Delayed Fluorescence: Analysis by Marcus-Hush Theory Including Reorganization Energy

Illia E. Serdiuk,* Michał Mońka, Karol Kozakiewicz, Beata Liberek, Piotr Bojarski, Soo Young Park

*Correspondence e-mail: illia.serdiuk@ug.edu.pl

Table of contents

Determination of experimental photophysical parameters Page S2
Table S1. Photoluminescent and photophysical parameters in various media Page S3

Analysis of molecular orbitals and nature of electronically excited states Page S4

Table S2. Computational data for various rotational isomers is the ^{3}CT-state geometry and rates of the ^{3}CT→^{1}CT transition Page S6

Table S3. Rates of ISC and rISC as statistical sums for rotamers for all investigated media Page S7

Prediction of the rate of radiative deactivation Page S8

Table S4. TD-DFT predicted energy ($\lambda_{S1\text{-}S0}$) and oscillator strengths (f) of S_1-S_0 transition, and calculated radiative deactivation constants (k_r) for various dihedral angles. Page S8

Figure S1. Dependence of the TD-DFT predicted energy gaps between singlet and triplet states on the dihedral angle between donor and acceptor fragments Page S8

Theoretical model including rotational isomers and vibrationally-enhanced SOC Page S9

Prediction of rISC rates. ^{3}CT→^{1}CT transition. Page S11

Prediction of rISC rates. ^{3}LE→^{1}CT transition. Page S12

Prediction of ISC rates. ^{1}CT→^{3}CT transition. Page S14

Prediction of ISC rates. ^{1}CT→^{3}LE transition. Page S14

Determination of photophysical parameters

Intensity weighted mean lifetime values were used for polyexponential decays of prompt (PF) or delayed (DF) fluorescence. The ratio of DF and PF quantum yields ($\varphi_{DF}/\varphi_{PF}$) was determined as following:

$$\frac{\varphi_{DF}}{\varphi_{PF}} = \frac{A_{DF}\tau_{DF}}{A_{PF}\tau_{PF}}, \tag{S1}$$

where A_{DF} and A_{PF} are pre-exponential factors of prompt and delayed fluorescence lifetimes, respectively; τ_{PF} and τ_{DF} are lifetimes of prompt and delayed fluorescence, respectively. Rate constants of radiative (k_r) and nonradiative (k_{nr}) decay, and intersystem crossing (k_{ISC}) were given by equations:

$$k_r = \frac{\varphi_{PF}}{\tau_{PF}}, \tag{S2}$$

$$k_{ISC} = \frac{\varphi_{DF}}{\tau_{PF}\varphi}, \tag{S3}$$

$$k_{nr} = \frac{1}{\tau_{PF}} - (k_r + k_{ISC}), \tag{S4}$$

where φ is PLQY ($\varphi_{DF} + \varphi_{PF}$).

Further, the quantum yields of ISC and rISC were calculated as

$$\varphi_{ISC} = k_{ISC}\tau_{PF}, \tag{S5}$$

$$\varphi_{rISC} = \frac{1 - \varphi_{PF}/\varphi}{\varphi_{ISC}}. \tag{S6}$$

And finally, rate constant of rISC (k_{rISC}) was calculated as

$$k_{rISC} = \frac{\varphi_{rISC}}{\tau_{DF}}(\varphi/\varphi_{PF}). \tag{S7}$$

Photophysical parameters are summarized below in Table S1.

Table S1. Photoluminescent and photophysical parameters in various media*

Solvent	PL_{max} [nm]	PLQY [%]	E_{1CT} (PL_{onset}) [nm]	E_{1CT} (PL_{onset}) [eV]	E_{3CT} [eV]	$\Delta E_{1CT\text{-}3LE}$ [eV]	$\Delta E_{1CT\text{-}3CT}$ [eV]	$\varphi_{DF}/\varphi_{PF}$	τ_{PF} [ns]	τ_{DF} [µs]	k_r [10^7 s^{-1}]	k_{nr} [10^7 s^{-1}]	k_{ISC} [10^7 s^{-1}]	k_{rISC} [10^7 s^{-1}]
Hexane	459	71	426	2.91	2.89	0.094	0.024[a]	1.1	15.4	32.0	2.2	0.9	3.4	0.007
Benzene-hexane (1/2)	480	60	436	2.84	2.83	0.026	0.019[a]	0.5	21.3	8.4	1.9	1.3	1.5	0.017
Benzene-hexane (1/1)	488	68	443	2.80	2.78	-0.022	0.016[a]	0.8	23.1	5.8	1.7	0.8	1.9	0.030
5% Acetone in hexane	522	100	451	2.75	2.73	-0.069	0.014[a]	1.2	31.9	6.1	1.4	0.0	1.7	0.036
Benzene	507	100	455	2.72	2.71	-0.095	0.013[a]	1.5	25.1	4.5	1.6	0.0	2.4	0.055
10% Acetone in hexane	543	73	465	2.67	2.66	-0.15	0.0115[b]	1.5	24.8	2.1	1.2	0.4	2.4	0.12
15% Acetone in hexane	561	63	474	2.62	2.61	-0.20	0.0084[b]	1.3	22.7	1.4	1.2	0.7	2.5	0.16
Chlorobenzene	550	78	482	2.57	2.56	-0.25	0.0088[b]	1.6	28.3	1.7	1.0	0.3	2.2	0.16
25% Acetone in hexane	578	38	489	2.54	2.53	-0.28	0.0064[b]	0.9	18.9	0.8	1.1	1.8	2.5	0.23
o-Dichlorobenzene	573	52	495	2.51	2.50	-0.31	0.0066[c]	1.1	26.7	1.0	0.91	0.8	2.0	0.21
75% Acetone in hexane	619	9.8	517	2.40	2.39	-0.42	0.0045[b]	0.3	8.7	0.4	0.85	7.8	2.8	0.35
Acetone	638	5.2	526	2.36	2.35	-0.46	0.0042[b]	0.2	5.8	0.3	0.74	13.4	3.1	0.38

*PL_{max} and PLQY – photoluminescence maximum and quantum yield, respectively;
E_{1CT} – energy of the ^{1}CT state obtained as onset of photoluminescence spectra;
E_{3CT} – energy of the ^{3}CT state calculated using E_{1CT} and $\Delta E_{1CT\text{-}3CT}$;
$\Delta E_{1CT\text{-}3LE}$ – energy gap between ^{1}CT and ^{3}LE states obtained from the onsets of respective emissions; negative values indicate that $E_{1CT} < E_{3LE}$.
$\Delta E_{1CT\text{-}3CT}$ – energy gap between ^{1}CT and ^{3}CT states obtained from Marcus-Hush equation and experimental k_{rISC} values corresponding to the dihedral angle of 90° (see text).
$\varphi_{DF}/\varphi_{PF}$ – ratio of quantum yields of delayed (DF) and prompt (PF) fluorescence;
τ_{PF}, τ_{DF} – lifetimes of PF and DF, respectively;
k_{ISC}, k_{rISC}, k_r, k_{nr} – rates of intersystem crossing, reverse intersystem crossing, radiative and nonradiative deactivation, respectively.
[a] Values obtained by extrapolation of the $\Delta E_{1CT\text{-}3CT} - E_{1CT}$ dependence. [b] Values obtained using Marcus-Hush equation and experimental k_{rISC} values. [c] Value predicted by TD-DFT calculations.

Analysis of molecular orbitals and nature of electronically excited states

The nature of states was assigned based on the orbitals involved in respective transitions available from single-point calculations using optimal geometry in corresponding state. Thus, based on the optimized geometry of T_1-state, calculated highest occupied (HOMO) and lowest unoccupied molecular orbitals (LUMO) involved in the T_1-S_0 transition are localized on separate fragments: 9,9-dimethyl-9,10-dihydroacridine (DMAC) and phenyl-*s*-triazine, respectively (Scheme S1). The T_1 state was thus assigned as a ^{3}CT state. The same conclusion was made for the S_1 state (^{1}CT).

Molecular orbitals, both HOMO and LUMO, involved in the T_2-S_0 transition calculated using the optimized geometry of T_2-state, are localized on the phenyl-*s*-triazine fragment (Scheme S1). Therefore, the T_2 state was assigned as a ^{3}LE state. Molecular orbitals involved in the T_3-S_0 transition calculated using the optimized geometry of T_3-state, are localized on the *N*-phenyl-DMAC fragment. The T_3 state was thus assigned as a second ^{3}LE state.

Opt. geometry	Assigned	Orbitals involved: HOMO / LUMO
S_1 and T_1*	CT	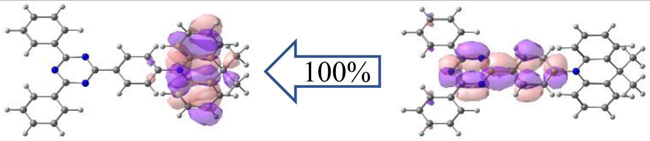
T_2	^{3}LE	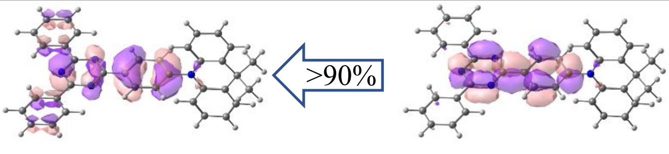
T_3	^{3}LE	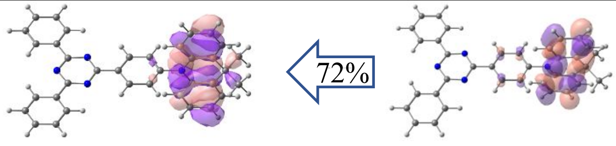 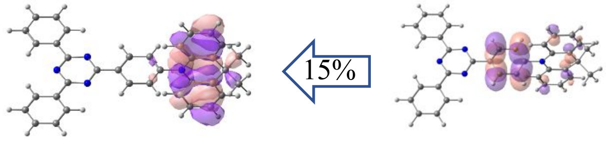

*geometries of the ^{1}CT and ^{3}CT states are almost identical so only the ^{1}CT one is shown.

Scheme S1. Molecular orbitals involved in the electronic trasitions of DMAC-TRZ

Experimentally, the CT nature of S_1 state was confirmed by the strong positive solvatofluorochromism (Figure 1, the main text). As was reported previously,[1] at 78 K, the phosphorescence spectra of DMAC-TRZ and its derivatives are independent of solvent polarity in the range of low polarities. Such phosphorescence thus originates from the triplet state of LE nature. In DMAC-TRZ, its maximum near 484 nm correlates well with the TDDFT calculated T_2-S_0 transition energy involving orbitals localized on the phenyl-*s*-

etriazine fragment. Such ^{3}LE-state energy determined experimentally as onset of phosphorescence spectrum in methylcyclohexane equals 2.82 eV (440 nm). The $\Delta E_{1CT\text{-}3LE}$ values in various solvents were thus estimated using the E_{1CT} values form the solvatofluorochromic measurements and the E_{3LE} value mentioned above. The emission from ^{3}CT-state was not registered experimentally in these investigations.

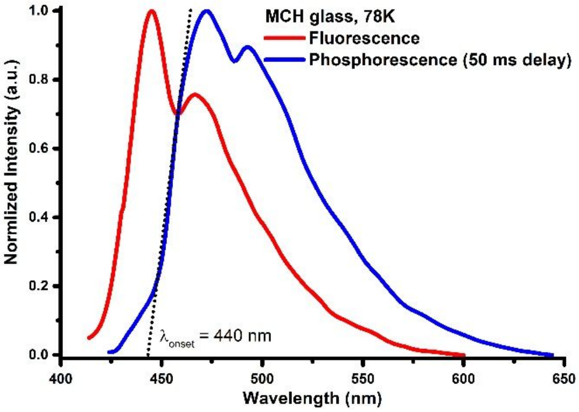

Table S2. Computational data for various rotational isomers is the ^{3}CT-state geometry and rates of the ^{3}CT→^{1}CT transition*

Dihedral Angle [°]	$V_{3CT\text{-}1CT}$ [cm^{-1}]	$\Delta E_{1CT\text{-}3CT}$ [eV]	$\lambda_{1CT\text{-}3CT}$ [eV]	$k_{3CT\rightarrow 1CT}$ [10^{7} s^{-1}]	μ [%]	$\mu \cdot k_{3CT\rightarrow 1CT}$ [10^{7} s^{-1}]
90	0.00	0.0066	0.0066	0.0000	5.20	0.0000
89 and 91	0.01	0.0068	0.0068	0.0224	10.39	0.0023
88 and 92	0.02	0.0076	0.0076	0.0824	10.25	0.0084
87 and 93	0.03	0.0089	0.0089	0.1631	10.07	0.0164
86 and 94	0.04	0.0107	0.0107	0.2459	9.80	0.0241
85 and 95	0.05	0.0130	0.0130	0.3186	9.46	0.0302
84 and 96	0.06	0.0159	0.0159	0.3723	9.05	0.0337
83 and 97	0.07	0.0192	0.0192	0.4042	8.56	0.0346
82 and 98	0.07	0.0230	0.0230	0.3187	8.02	0.0256
81 and 99	0.08	0.0273	0.0273	0.3231	7.42	0.0240
80 and 100	0.09	0.0320	0.0320	0.3143	6.79	0.0213
75 and 105	0.13	0.0621	0.0621	0.1458	3.55	0.0052
70 and 110	0.16	0.1012	0.1012	0.0378	1.22	0.0005
65 and 115	0.19	0.1467	0.1467	0.0075	0.21	0.0000
**Sum**						**0.226**

*$V_{3CT\text{-}1CT}$ – SOCME value for the ^{3}CT→^{1}CT transition; μ – statistical weight of a rotational isomer at room temperature calculated using relative energies of isomers in the ^{3}CT state listed in Table S5.

Table S3. Rates of ISC and rISC as statistical sums for rotamers for all investigated media*

Solvent	χ_{3CT} [%]	χ_{3LE} [%]	ISC [10^7 s^{-1}]			rISC [10^7 s^{-1}]				
			$k_{1CT\rightarrow 3CT}$	$k_{1CT\rightarrow 3LE}$	$k_{1CT\rightarrow 3CT}$ + $k_{1CT\rightarrow 3LE}$	$k_{3CT\rightarrow 1CT}$	$k_{3LE\rightarrow 1CT}$	$\chi_{3CT}k_{3CT\rightarrow 1CT}$	$\chi_{3LE}k_{3LE\rightarrow 1CT}$	$\chi_{3CT}k_{3CT\rightarrow 1CT}$ + $\chi_{3LE}k_{3LE\rightarrow 1CT}$
Hexane	5.6	94.4	0.26	1.27	1.53	0.023	0.034	$1.3\cdot10^{-3}$	0.032	0.033
Benzene-hexane (1/2)	42.8	57.2	0.28	0.39	0.67	0.039	0.15	0.017	0.085	0.102
Benzene-hexane (1/1)	81.4	18.6	0.29	0.15	0.44	0.053	0.38	0.043	0.070	0.114
Acetone-hexane (5%)	96.3	3.7	0.31	$5.6\cdot10^{-2}$	0.37	0.071	0.87	0.068	0.032	0.100
Benzene	98.6	1.4	0.32	$3.1\cdot10^{-2}$	0.35	0.082	1.32	0.081	0.053	0.134
Acetone-hexane (10%)	99.8	0.18	0.35	$7.9\cdot10^{-3}$	0.36	0.111	2.95	0.111	0.014	0.124
Acetone-hexane (15%)	100	$2.8\cdot10^{-2}$	0.38	$2.1\cdot10^{-3}$	0.38	0.142	5.55	0.142	$3.7\cdot10^{-3}$	0.146
Chlorobenzene	100	$5.1\cdot10^{-3}$	0.40	$4.8\cdot10^{-4}$	0.40	0.172	9.49	0.172	$1.1\cdot10^{-3}$	0.173
Acetone-hexane (25%)	100	$1.3\cdot10^{-3}$	0.43	$1.9\cdot10^{-4}$	0.43	0.202	12.5	0.202	$3.3\cdot10^{-4}$	0.202
o-Dichlorobenzene	100	$3.9\cdot10^{-4}$	0.46	$7.4\cdot10^{-5}$	0.46	0.229	15.9	0.229	$1.2\cdot10^{-4}$	0.229
Acetone-hexane (75%)	100	$6.7\cdot10^{-6}$	0.56	$1.8\cdot10^{-6}$	0.56	0.338	28.8	0.338	$2.5\cdot10^{-6}$	0.338
Acetone	100	$1.4\cdot10^{-6}$	0.58	$3.6\cdot10^{-7}$	0.58	0.387	31.8	0.387	$4.8\cdot10^{-7}$	0.387

*χ_{3CT} and χ_{3LE} – molar fractions of molecules coexisting in ^{3}CT and ^{3}LE states at 298.15 K, calculated using Boltzmann distribution law and energies of the respective triplet states listed in Table S1.

$k_{1CT\rightarrow 3LE}$ and $k_{3LE\rightarrow 1CT}$ calculated using λ_{solv} = 0.3 eV (Table S9 and S10)

Prediction of the rate of radiative deactivation (k_r). The rate of radiative deactivation was obtained as a statistical sum of respective values for each rotational isomer calculated using Strickler-Berg law:[2,3]

$$k_r = \sum_{\theta} \frac{0.668 \cdot f(\theta) \cdot n^2}{\lambda^2_{S1-S0}(\theta) \cdot 10^7} \quad \text{(S8)},$$

where $\lambda_{S1\text{-}S0}(\theta)$ and $f(\theta)$ are wavelength in nanometer and oscillator strength of S_1-S_0 transition for each rotational isomer, respectively; n is refractive index of *o*-dichlorobenzene (n = 1.551).

Table S4. TD-DFT predicted energy ($\lambda_{S1\text{-}S0}$) and oscillator strengths (f) of S_1-S_0 transition, and calculated radiative deactivation constants (k_r) for various dihedral angles*

Dihedral Angle θ [°]	$\lambda_{S1\text{-}S0}$ [nm]	f	k_r [10^7 s^{-1}]	μ [%]	$\mu \cdot k_r$ [10^7 s^{-1}]
90	611.49	0.0000	0.000	7.37	0.0000
89 and 91	611.22	0.0002	0.009	14.2	0.0012
88 and 92	610.98	0.0008	0.034	13.6	0.0047
87 and 93	610.61	0.0018	0.078	12.7	0.0099
86 and 94	610.13	0.0031	0.134	11.5	0.0154
85 and 95	609.49	0.0049	0.212	10.2	0.0215
84 and 96	608.72	0.0070	0.304	8.70	0.0264
83 and 97	607.77	0.0096	0.418	7.23	0.0302
82 and 98	606.76	0.0125	0.546	5.84	0.0319
81 and 99	605.58	0.0158	0.692	4.57	0.0316
80 and 100	604.29	0.0194	0.854	3.48	0.0297
75 and 105	596.13	0.0428	1.935	0.56	0.0109
70 and 110	585.64	0.0740	3.467	0.04	0.0015
65 and 115	573.47	0.1124	5.492	0.001	0.0001
**Sum**					**0.215**

*μ – statistical weight of a rotational isomer in ^{1}CT-state at room temperature calculated using relative energies of isomers listed in Table S5.

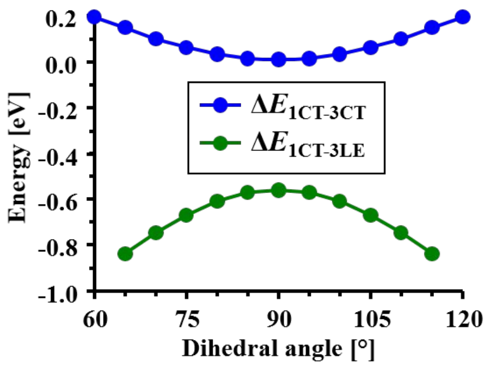

Figure S1. Dependence of the TD-DFT predicted energy gaps between singlet and triplet states on the dihedral angle between donor and acceptor fragments

Theoretical model including rotational isomers and vibrationally-enhanced SOC

Corrections for various polarity. Alignment of the potential curves of ^{1}CT, ^{3}CT, and ^{3}LE states predicted on the TD-TDF/B3LYP level of theory is in excellent correlation with the experimental data in the media of relatively high polarity (like *o*-dichlorobenzene and 75% mixture of acetone in hexane). To mimic less and more polar medium, the energies of ^{1}CT and ^{3}CT states were corrected for each media according to the procedure below.

Unconstrained geometry optimizations of DMAC-TRZ were conducted for various excited states. The nature of each excited state was established by the analysis of molecular orbitals involved in respective transitions. The $\Delta E_{1CT\text{-}3CT}$ and $\Delta E_{1CT\text{-}3LE}$ values were calculated using electronic energies (E(TD-DFT)) of respective states in their energetic minima (Table S5), verified by the absence of imaginary (negative) vibrational frequencies. Next, starting with the optimized geometry in each excited state, the value of dihedral angle between DMAC and phenyl ring of acceptor fragment was scanned with a 1 degree step from 89° to 60°. Energies of respective states were calculated by single-point calculations. The $\Delta E_{1CT\text{-}3CT}$ and $\Delta E_{1CT\text{-}3LE}$ values for each rotational isomer were calculated using thus obtained energies.

Table S5. Energies of the excited states and gaps between them.

						Corrected for hexane	
Dihedral Angle [°]	$E_{1CT}{}^{1CT}$ [au]	$E_{3CT}{}^{3CT}$ [au]	$E_{3LE}{}^{3LE}$ [au]	$\Delta E_{1CT\text{-}3CT}$ [eV]	$\Delta E_{1CT\text{-}3LE}$ [eV]	$\Delta E_{1CT\text{-}3CT}$ [eV]	$\Delta E_{1CT\text{-}3LE}$ [eV]
90	-1607.76452079	-1607.76476132	-1607.74397545	0.0065	-0.559	0.024	0.094
85	-1607.76417036	-1607.76467305	-1607.74347908	0.0137	-0.563	0.049	0.090
80	-1607.76315795	-1607.76435939	-1607.74202884	0.0327	-0.575	0.118	0.078
75	-1607.76143695	-1607.76374597	-1607.73979476	0.0628	-0.589	0.227	0.064
70	-1607.75899058	-1607.76273759	-1607.73691709	0.1020	-0.601	0.368	0.052
65	-1607.75567303	-1607.76121844	-1607.73334284	0.1509	-0.608	0.545	0.045

Table S6. Vertical transition energies of DMAC-TRZ calculated using PCM

Medium	Vertical transition energy [nm]	
	S_0-S_1	S_1-S_0
Vacuum	516	612
Chloroform	500	597
Formamide	496	593

DFT fails to predict medium polarity influence on the spectral properties of D-A emitters. Polarized continuum model, most extensively used for this purpose predicts incorrectly the solvent effect. (Table S6). To mimic medium of different polarity, minimum ^{1}CT energy, energies of rotational isomers in ^{1}CT state as well as all vertical transitions involving ^{1}CT state were corrected by a correction factor C, obtained using experimental $\Delta E_{1CT\text{-}3LE}$ value (Table S1) for each medium:

$$C = E_{3LE}{}^{3LE} - E_{1CT}{}^{1CT} + \Delta E_{1CT\text{-}3LE} \quad \text{(S9)},$$

where $E_{3LE}{}^{3LE}$ and $E_{3LE}{}^{3LE}$ are the energies of respective states in their optimized geometries from Table S5 (dihedral angle 90°). Obtained C values are listed below (Table S7):

Table S7. Correction factors for various media

**Solvent**	**$\Delta E_{1CT\text{-}3LE}$ [eV]**	***C***
Hexane	0.094	0.65
Benzene-hexane (1/2)	0.026	0.58
Benzene-hexane (1/1)	-0.022	0.54
Acetone-hexane (5%)	-0.069	0.49
Benzene	-0.095	0.46
Acetone-hexane (10%)	-0.15	0.41
Acetone-hexane (15%)	-0.20	0.36
Chlorobenzene	-0.25	0.31
Acetone-hexane (25%)	-0.28	0.28
o-Dichlorobenzene	-0.31	0.25
Acetone-hexane (75%)	-0.42	0.14
Acetone	-0.46	0.10

For each medium, the $\Delta E_{1CT\text{-}3LE}$ values at various dihedral angles (θ) were calculated as follows:

$$\text{“}\Delta E_{1CT\text{-}3LE}(\theta)\text{”} = (E_{1CT}{}^{1CT}(\theta) + C) - E_{3LE}{}^{3LE}(\theta)\text{”} \quad \text{(S10)},$$

The example of such calculations for hexane is listed in Table S5.

As the $\Delta E_{1CT\text{-}3CT}$ value changes also with the change of dihedral angle, for each medium, its value for each rotational isomer “$\Delta E_{1CT\text{-}3CT}{}^{Solvent}(\theta)$” was calculated proportional to that at 90°. In the example with hexane:

$$\text{“}\Delta E_{1CT\text{-}3CT}{}^{Hexane}(\theta)\text{”} = \text{“}\Delta E_{1CT\text{-}3CT}(\theta)\text{”} \cdot \text{“}\Delta E_{1CT\text{-}3CT}{}^{Hexane}\text{”}/\ \text{“}\Delta E_{1CT\text{-}3CT}\text{”} \quad \text{(S11)},$$

where

"$\Delta E_{1CT\text{-}3CT}(\theta)$" – computational value for respective dihedral angle from Table S5;

"$\Delta E_{1CT\text{-}3CT}$" – computational value for 90° dihedral angle from Table S5;

"$\Delta E_{1CT\text{-}3CT}^{Hexane}$" – experimental value listed for each medium in Table S1.

The example of such calculations for hexane is listed in Table S5.

Reorganization energies of the ^{3}LE→^{1}CT and ^{1}CT→^{3}LE transitions (Figure 3) were calculated as follows

$$\lambda_{3LE\text{-}1CT}(\theta) = E_{1CT}^{3LE}(\theta) - E_{1CT}^{1CT}(\theta) \quad \text{(S12)},$$

$$\lambda_{1CT\text{-}3LE}(\theta) = E_{3LE}^{1CT}(\theta) - E_{3LE}^{3LE}(\theta) \quad \text{(S13)},$$

where

$E_{1CT}^{3LE}(\theta)$ – TD-DFT energy of the ^{1}CT state at the ^{3}LE geometry at given dihedral angle value θ; $E_{3LE}^{1CT}(\theta)$ – TD-DFT energy of the ^{3}LE state at the ^{1}CT geometry at given dihedral angle value θ. These values are listed in Table S8.

Table S8. Energies of states in non-optimal geometries and reorganization energies

**Dihedral Angle [°]**	E_{1CT}^{3LE} **[au]**	E_{3LE}^{1CT} **[au]**	$\lambda_{1CT\text{-}3LE}$ **[eV]**	$\lambda_{3LE\text{-}1CT}$ **[eV]**
90	-1607.756846	-1607.737375	0.1796	0.2088
85	-1607.756471	-1607.736974	0.1770	0.2095
80	-1607.755313	-1607.735792	0.1697	0.2135
75	-1607.753388	-1607.733989	0.1580	0.2190
70	-1607.750780	-1607.731688	0.1423	0.2234
65	-1607.747374	-1607.728820	0.1231	0.2258

Prediction of rISC rates. ^{3}CT→^{1}CT transition.

Parameters for calculations of ^{3}CT→^{1}CT transition rate for various rotational isomers using Marcus-Hush equation and the B3LYP predicted $\Delta E_{1CT\text{-}3CT}$ values are presented in Table S2. For media with $E_{1CT} \leq 2.67$ eV, experimental k_{rISC} was used for calculation of $\Delta E_{1CT\text{-}3CT}$ using Marcus-Hush equation and assuming $\Delta E_{1CT\text{-}3CT} = \lambda_{1CT\text{-}3CT}$. For less polar media, $\Delta E_{1CT\text{-}3CT}$ were obtained by extrapolation (Figure 2H in the main text). Reconstructed k_{rISC} values for all media are shown in Table S3.

Prediction of rISC rates. $^3LE{\rightarrow}^1CT$ transition. Examples of calculations of ^{3}LE→^{1}CT transition rates for various rotational isomers using Marcus-Hush equation are presented in Table S9.

Table S9. Calculation of the ^{3}LE→^{1}CT transition parameters*

Hexane, $\Delta E_{1CT\text{-}3LE}$ = 0.094 eV.

**Dihedral Angle [°]**	**$\Delta E_{1CT\text{-}3LE}$ [eV]**	**$\lambda_{3LE\text{-}1CT}$ [eV]**	**μ [%]**	**λ_{solv} = 0.2 eV**		**λ_{solv} = 0.3 eV**	
				**$k_{3LE\rightarrow1CT}$ [10^7 s^{-1}]**	**$\mu{\cdot}k_{3LE\rightarrow1CT}$ [10^7 s^{-1}]**	**$k_{3LE\rightarrow1CT}$ [10^7 s^{-1}]**	**$\mu{\cdot}k_{3LE\rightarrow1CT}$ [10^7 s^{-1}]**
90	0.094	0.2088	40.6	0.092	0.037	0.032	0.013
85 and 95	0.090	0.2095	48.0	0.098	0.047	0.035	0.017
80 and 100	0.078	0.2135	10.3	0.115	0.012	0.040	0.004
75 and 105	0.064	0.2190	1.0	0.131	0.001	0.045	0.000
70 and 110	0.052	0.2234	0.0	0.134	0.000	0.046	0.000
65 and 115	0.045	0.2258	0.0	0.120	0.000	0.041	0.000
					**0.098**		**0.034**

Benzene-hexane (1:2), $\Delta E_{1CT\text{-}3LE}$ = 0.026 eV.

**Dihedral Angle [°]**	**$\Delta E_{1CT\text{-}3LE}$ [eV]**	**$\lambda_{3LE\text{-}1CT}$ [eV]**	**μ [%]**	**λ_{solv} = 0.2 eV**		**λ_{solv} = 0.3 eV**	
				**$k_{3LE\rightarrow1CT}$ [10^7 s^{-1}]**	**$\mu{\cdot}k_{3LE\rightarrow1CT}$ [10^7 s^{-1}]**	**$k_{3LE\rightarrow1CT}$ [10^7 s^{-1}]**	**$\mu{\cdot}k_{3LE\rightarrow1CT}$ [10^7 s^{-1}]**
90	0.026	0.2088	40.6	0.419	0.170	0.142	0.058
85 and 95	0.022	0.2095	48.0	0.443	0.213	0.150	0.072
80 and 100	0.010	0.2135	10.3	0.497	0.051	0.169	0.017
75 and 105	-0.004	0.2190	1.0	0.540	0.005	0.183	0.002
70 and 110	-0.016	0.2234	0.0	0.533	0.000	0.181	0.000
65 and 115	-0.023	0.2258	0.0	0.467	0.000	0.159	0.000
**Sum**					**0.440**		**0.149**

Benzene-hexane (1:1) mixture, $\Delta E_{1CT\text{-}3LE}$ = –0.022 eV.

**Dihedral Angle [°]**	**$\Delta E_{1CT\text{-}3LE}$ [eV]**	**$\lambda_{3LE\text{-}1CT}$ [eV]**	**μ [%]**	**λ_{solv} = 0.2 eV**		**λ_{solv} = 0.3 eV**	
				**$k_{3LE\rightarrow1CT}$ [10^7 s^{-1}]**	**$\mu{\cdot}k_{3LE\rightarrow1CT}$ [10^7 s^{-1}]**	**$k_{3LE\rightarrow1CT}$ [10^7 s^{-1}]**	**$\mu{\cdot}k_{3LE\rightarrow1CT}$ [10^7 s^{-1}]**
90	-0.022	0.2088	40.6	1.073	0.436	0.364	0.148
85 and 95	-0.026	0.2095	48.0	1.123	0.539	0.381	0.183
80 and 100	-0.038	0.2135	10.3	1.226	0.127	0.418	0.043
75 and 105	-0.052	0.2190	1.0	1.292	0.013	0.444	0.004
70 and 110	-0.064	0.2234	0.0	1.243	0.001	0.430	0.000
65 and 115	-0.071	0.2258	0.0	1.074	0.000	0.373	0.000
**Sum**					**1.115**		**0.379**

Table S9 continued.

Benzene, $\Delta E_{1CT\text{-}3LE}$ = –0.095 eV.

**Dihedral Angle [°]**	**$\Delta E_{1CT\text{-}3LE}$ [eV]**	**$\lambda_{3LE\text{-}1CT}$ [eV]**	**μ [%]**	**λ_{solv} = 0.2 eV**		**λ_{solv} = 0.3 eV**	
				**$k_{3LE\to1CT}$ [10^7 s^{-1}]**	**$\mu \cdot k_{3LE\to1CT}$ [10^7 s^{-1}]**	**$k_{3LE\to1CT}$ [10^7 s^{-1}]**	**$\mu \cdot k_{3LE\to1CT}$ [10^7 s^{-1}]**
90	-0.095	0.2088	40.6	3.627	1.474	1.281	0.520
85 and 95	-0.099	0.2095	48.0	3.745	1.798	1.327	0.637
80 and 100	-0.111	0.2135	10.3	3.934	0.407	1.411	0.146
75 and 105	-0.125	0.2190	1.0	3.966	0.038	1.443	0.014
70 and 110	-0.137	0.2234	0.0	3.681	0.002	1.357	0.001
65 and 115	-0.144	0.2258	0.0	3.112	0.000	1.157	0.000
**Sum**					**3.719**		**1.318**

Acetone, $\Delta E_{1CT\text{-}3LE}$ = –0.46 eV.

**Dihedral Angle [°]**	**$\Delta E_{1CT\text{-}3LE}$ [eV]**	**$\lambda_{3LE\text{-}1CT}$ [eV]**	**μ [%]**	**λ_{solv} = 0.2 eV**		**λ_{solv} = 0.3 eV**	
				**$k_{3LE\to1CT}$ [10^7 s^{-1}]**	**$\mu \cdot k_{3LE\to1CT}$ [10^7 s^{-1}]**	**$k_{3LE\to1CT}$ [10^7 s^{-1}]**	**$\mu \cdot k_{3LE\to1CT}$ [10^7 s^{-1}]**
90	-0.460	0.2088	40.6	35.56	14.44	32.42	13.17
85 and 95	-0.464	0.2095	48.0	34.54	16.59	31.94	15.34
80 and 100	-0.476	0.2135	10.3	30.99	3.20	29.68	3.07
75 and 105	-0.490	0.2190	1.0	26.35	0.26	26.19	0.25
70 and 110	-0.502	0.2234	0.0	21.20	0.01	21.75	0.01
65 and 115	-0.509	0.2258	0.0	16.44	0.00	17.21	0.00
**Sum**					**34.50**		**31.84**

* μ – statistical weight of a rotational isomer in ^{3}LE-state at room temperature calculated using relative energies of isomers listed in Table S5.

Prediction of ISC rates. $^1CT{\rightarrow}^3CT$ transition. Rates of the ^{1}CT→^{3}CT transition in various media were calculated as statistical sums for various rotational isomers using Marcus-Hush equation. The used $\Delta E_{1CT\text{-}3CT}$ values were calculated using equation S11 and are listed in Table S1. SOCME values are listed in Table S2. The $\lambda_{1CT\text{-}3CT}$ values were assumed equal to the $\Delta E_{1CT\text{-}3CT}$ ones. Thus obtained $k_{1CT\rightarrow 3CT}$ values for all media are listed in Table S3.

Prediction of ISC rates. $^1CT{\rightarrow}^3LE$ transition.

Examples of calculations of the ^{1}CT→^{3}LE transition rates for various rotational isomers using Marcus-Hush equation are presented in Table S10.

Table S10. Calculation of the ^{1}CT→^{3}LE transition parameters*

Hexane, $\Delta E_{1CT\text{-}3LE}$ = 0.094 eV.

Dihedral Angle [°]	$\Delta E_{3LE\text{-}1CT}$ [eV]	$\lambda_{1CT\text{-}3LE}$ [eV]	μ [%]	λ_{solv} = 0.2 eV		λ_{solv} = 0.3 eV	
				$k_{1CT\rightarrow 3LE}$ [10^7 s^{-1}]	$\mu{\cdot}k_{1CT\rightarrow 3LE}$ [10^7 s^{-1}]	$k_{1CT\rightarrow 3LE}$ [10^7 s^{-1}]	$\mu{\cdot}k_{1CT\rightarrow 3LE}$ [10^7 s^{-1}]
90	-0.094	0.1796	34.1	3.67	1.25	1.29	0.441
85 and 95	-0.090	0.1731	47.0	3.71	1.74	1.30	0.612
80 and 100	-0.078	0.1538	16.1	3.30	0.53	1.14	0.184
75 and 105	-0.064	0.1281	2.6	3.42	0.089	1.16	0.030
70 and 110	-0.052	0.1007	0.2	3.37	$6.6{\cdot}10^{-3}$	1.13	$2.2{\cdot}10^{-3}$
65 and 115	-0.045	0.0746	0.0	3.32	$1.9{\cdot}10^{-4}$	1.09	$6.4{\cdot}10^{-5}$
**Sum**					**3.62**		**1.27**

Benzene, $\Delta E_{1CT\text{-}3LE}$ = -0.095 eV.

Dihedral Angle [°]	$\Delta E_{3LE\text{-}1CT}$ [eV]	$\lambda_{1CT\text{-}3LE}$ [eV]	μ [%]	λ_{solv} = 0.2 eV		λ_{solv} = 0.3 eV	
				$k_{1CT\rightarrow 3LE}$ [10^7 s^{-1}]	$\mu{\cdot}k_{1CT\rightarrow 3LE}$ [10^7 s^{-1}]	$k_{1CT\rightarrow 3LE}$ [10^7 s^{-1}]	$\mu{\cdot}k_{1CT\rightarrow 3LE}$ [10^7 s^{-1}]
90	0.095	0.1796	34.1	0.092	0.0314	0.032	0.0111
85 and 95	0.099	0.1731	47.0	0.089	0.0421	0.032	0.0149
80 and 100	0.111	0.1538	16.1	0.070	0.0113	0.025	$4.1{\cdot}10^{-3}$
75 and 105	0.125	0.1281	2.6	0.061	$1.6{\cdot}10^{-3}$	0.023	$5.9{\cdot}10^{-4}$
70 and 110	0.137	0.1007	0.2	0.051	$9.9{\cdot}10^{-5}$	0.019	$3.8{\cdot}10^{-5}$
65 and 115	0.144	0.0746	0.0	0.043	$2.5{\cdot}10^{-6}$	0.017	$9.9{\cdot}10^{-7}$
**Sum**					**0.086**		**0.031**

Table S10 continued

Acetone, $\Delta E_{1CT\text{-}3LE}$ = -0.46 eV.

Dihedral Angle [°]	$\Delta E_{3LE\text{-}1CT}$ [eV]	$\lambda_{1CT\text{-}3LE}$ [eV]	μ [%]	λ_{solv} = 0.2 eV		λ_{solv} = 0.3 eV	
				$k_{1CT\to3LE}$ [10^7 s^{-1}]	$\mu\cdot k_{1CT\to3LE}$ [10^7 s^{-1}]	$k_{1CT\to3LE}$ [10^7 s^{-1}]	$\mu\cdot k_{1CT\to3LE}$ [10^7 s^{-1}]
90	0.460	0.1796	34.1	$4.2\cdot10^{-7}$	$1.4\cdot10^{-7}$	$4.4\cdot10^{-7}$	$1.5\cdot10^{-7}$
85 and 95	0.464	0.1731	47.0	$3.4\cdot10^{-7}$	$1.6\cdot10^{-7}$	$3.8\cdot10^{-7}$	$1.8\cdot10^{-7}$
80 and 100	0.476	0.1538	16.1	$1.6\cdot10^{-7}$	$2.5\cdot10^{-8}$	$2.1\cdot10^{-7}$	$3.4\cdot10^{-8}$
75 and 105	0.490	0.1281	2.6	$6.5\cdot10^{-8}$	$1.7\cdot10^{-9}$	$1.1\cdot10^{-7}$	$2.9\cdot10^{-9}$
70 and 110	0.502	0.1007	0.2	$2.2\cdot10^{-8}$	$4.3\cdot10^{-11}$	$5.5\cdot10^{-8}$	$1.1\cdot10^{-10}$
65 and 115	0.509	0.0746	0.0	$7.7\cdot10^{-9}$	$4.5\cdot10^{-13}$	$2.9\cdot10^{-8}$	$1.7\cdot10^{-12}$
**Sum**					**$3.3\cdot10^{-7}$**		**$3.6\cdot10^{-7}$**

* μ – statistical weight of a rotational isomer in ^{1}CT-state at room temperature calculated using relative energies of isomers listed in Table S5.

Summary of the ISC and rISC rates as statistical sums for rotamers calculated according to the Marcus-Hush equation is presented in Table S3.

References

[1] Serdiuk, I. E.; Ryoo, C. H.; Kozakiewicz, K.; Mońka, M.; Liberek, B.; Park, S. Y. Twisted Acceptors in the Design of Deep-Blue TADF Emitters: Crucial Role of the Excited-State Relaxation on the Photophysics of Methyl Substituted *s*-Triphenyltriazine Derivatives. *J. Mater. Chem. C* **2020**, *8*, 6052-6062.

[2] Strickler, S. J.; Berg, R. A.. Relationship between absorption intensity and fluorescence lifetime of molecules. *J. Chem. Phys.* **1962**, *37*, 814–822.

[3] Mohanty, J.; Na, W. M. Refractive index effects on the oscillator strength and radiative decay rate of 2,3-diazabicyclo[2.2.2]oct-2-ene. *Photochem. Photobiol. Sci.* **2004**, *3*, 1026–1031.
